# Fuzzy-Based Microservice Resource Management Platform for Edge Computing in the Internet of Things

**DOI:** 10.3390/s21113800

**Published:** 2021-05-31

**Authors:** David Chunhu Li, Chiing-Ting Huang, Chia-Wei Tseng, Li-Der Chou

**Affiliations:** 1 Information Technology and Management Program, Ming Chuan University, Taoyuan City 333321, Taiwan; davidli@mail.mcu.edu.tw; 2 Department of Computer Science and Information Engineering, National Central University, Taoyuan City 320317, Taiwan; ginting@g.ncu.edu.tw (C.-T.H.); cwtseng@g.ncu.edu.tw (C.-W.T.)

**Keywords:** edge computing, fuzzy system, Internet of Things, microservice, resource management, scaling

## Abstract

Edge computing exhibits the advantages of real-time operation, low latency, and low network cost. It has become a key technology for realizing smart Internet of Things applications. Microservices are being used by an increasing number of edge computing networks because of their sufficiently small code, reduced program complexity, and flexible deployment. However, edge computing has more limited resources than cloud computing, and thus edge computing networks have higher requirements for the overall resource scheduling of running microservices. Accordingly, the resource management of microservice applications in edge computing networks is a crucial issue. In this study, we developed and implemented a microservice resource management platform for edge computing networks. We designed a fuzzy-based microservice computing resource scaling (FMCRS) algorithm that can dynamically control the resource expansion scale of microservices. We proposed and implemented two microservice resource expansion methods based on the resource usage of edge network computing nodes. We conducted the experimental analysis in six scenarios and the experimental results proved that the designed microservice resource management platform can reduce the response time for microservice resource adjustments and dynamically expand microservices horizontally and vertically. Compared with other state-of-the-art microservice resource management methods, FMCRS can reduce sudden surges in overall network resource allocation, and thus, it is more suitable for the edge computing microservice management environment.

## 1. Introduction

With the recent development of emerging technologies, the information industry is vigorously developing the integration of edge computing and cloud computing and the introduction of edge computing into the vertical field. The rapid development of the Internet of Things (IoT) has promoted the development of various flexible and decentralized computing architectures in the industry. In the edge–cloud computing architecture, edge computing plays a role in the local learning and filtering of data transmitted by a terminal device, while simultaneously sharing the computing and storage tasks of the cloud [1,2,3]. From the perspective of cloud computing to edge computing, the cloud platform can deploy various microservices to the edge computing network in accordance with the needs of a terminal device [4]. Edge computing exhibits the advantages of real-time operation, low latency, and low network cost, enabling it to share the cloud’s load; this capability has become an important consideration in the design of IoT system architecture [5,6]. 

Edge computing is a distributed network infrastructure that allows data to be preprocessed and analyzed closer to their source. Edge computing can be used in various applications, such as IoT, artificial intelligence, and big data. The microservice architecture involves the development of applications as a collection of small services, wherein each service can implement business functions, run its process, and communicate through HyperText Transfer Protocol (HTTP) application programming interfaces (APIs). Each microservice can be deployed, upgraded, expanded, and restarted independently of other services in the application [7]. Therefore, the integration of edge computing and microservices can provide a flexible and diverse application development architecture.

Microservices are functionally meaningful applications that are sufficiently small to focus on a specific business function or requirement [8,9]. Microservices are independent during either the service development or deployment phase; they are also easy to integrate into third-party application systems, enabling easy and flexible integration and automatic deployment [10,11]. Given the aforementioned advantages, various microservice applications [12,13,14] have been run extensively in cloud computing networks. However, edge computing has more limited resources than cloud computing, and thus edge computing networks have higher requirements for the overall resource scheduling for the running of microservices [15,16]. The smooth network communication of various containerized microservices must be ensured. Therefore, the resource management of microservice applications in edge computing networks is a crucial issue [17].

Edge computing microservice resource management is currently facing two major challenges [18,19]: (1) how to manage the computing resource requirements of various microservice programs effectively, and (2) how to determine the priority of processing various microservices based on available resources. To solve the first problem, several edge computing service management platforms have adopted an auto-scaling algorithm, which is the primary resource management method for cloud computing application services [20,21,22]. Auto-scaling includes three basic strategies: automatic, predictive, and event-reactive scaling [23]. During the automatic scaling of a scheduling program, the corresponding rules for system time and the resource deployment scale should be written in advance; thus, this technique can only be used in a network environment in which the number of service requirements and time conditions are stable. Predictive scaling requires additional learning costs, and it exhibits poor performance in emergencies. Although event-reactive scaling can respond in time to the current resource usage requirements, it cannot achieve the same performance as that which it displays in a cloud computing network when it is applied to a delay-sensitive edge computing network with limited computing resources. The second challenge primarily involves controlling the use of edge node resources [24,25,26]. The available resources are constantly changing in an edge network. In contrast with a cloud computing network, which has nearly unlimited computing resources, edge computing networks tend to limit the use of services due to their limited computing resources. Each service has a priority; thus, using an optimal method to determine the priority of processing services based on available resources is essential. To address these two challenges, in this study, we present the design of an edge computing fuzzy-based microservice resource management platform.

In traditional network service resource management methods, researchers usually design various computing resource management algorithms or mechanisms to manage the computing resources of the program. These management algorithms and mechanisms evaluate the resource expansion requirements of applications or microservices based on metrics such as the resources usage amount of the programs. Accordingly, the algorithms calculate that more or less computing resources are to be allocated to applications and microservices.

However, the amount of computing resources used or required is usually based on a vague vocabulary. In particular, the network topology of the edge computing network changes rapidly, and the operating status of the computing nodes and running microservices in the edge computing network also changes frequently. These factors have caused many ambiguities and uncertainties in microservice resource management requirements [27]. If these vague and uncertain requirements are used as input parameters for traditional resource expansion algorithms, people have to design very complex equations, algorithms, and system architectures to cope with managing complicated service resources in an edge computing network. Furthermore, complex algorithms require more computing resources and computing time. The complex resource management software system architecture also has the problems of difficulty in system development and poor scalability. These problems reduce the performance of traditional resource management algorithms and mechanisms in edge computing networks.

Fuzzy theory models the process of human subjective thinking or judgment. Designing fuzzy logic systems based on fuzzy theory does not require an accurate mathematical model of the controlled object. Instead, fuzzy sets are used for quantitative processing. Fuzzy theory has been widely used in various products that combine computers and human subjectivity [28]. Fuzzy theory makes the control mechanism and strategy easy to accept and understand.

Compared with traditional algorithmic systems, fuzzy logic systems are simple in design and easy to apply. The fuzzy logic system can simplify the complexity of system design and is especially suitable for non-linear, time-varying application systems. However, the fuzzy-based resource management method still has some shortcomings [29]. For instance, a comprehensive and systematic design method is needed to design a service resource management system based on fuzzy theory, and this is a challenge for designers. For different application fields, it is necessary to customize the design of fuzzy rules and the membership functions of fuzzy logic systems, and there is no unified standard approach. Moreover, the form of fuzzy rule bases and membership functions has a great influence on the performance of the fuzzy logic system, which also increases the difficulty of system tuning.

In this study, we use the advantages and characteristics of fuzzy theory to improve the resource management methods for edge computing network microservices. We focus on designing and implementing a prototype of an edge computing microservice resource management platform, which includes a fuzzy-based microservice computing resource scaling (FMCRS) algorithm. FMCRS represents the state of computing resources of microservices running on edge computing nodes in the form of fuzzy sets and evaluates the scaled extent of computing resources of microservices based on 27 fuzzy rules. The major contributions of this work are summarized as follows.

(1) In this study, we design and implement a prototype of an edge computing network microservice resource management platform. This platform can provide a solution to cloud computing service providers and edge computing system users for realizing the effective resource management of each microservice in an edge computing network. The system prototype designed in this work provides users of edge computing networks with a new option to meet the increasingly complex network application requirements.
(2) We design a fuzzy-based microservice computing resource scaling (FMCRS) algorithm that can dynamically control the resource expansion scale of microservices to meet their resource consumption requirements in a timely manner. FMCRS can ensure the lowest availability of various microservices.
(3) We apply the particle swarm optimization (PSO) algorithm to optimize the proposed fuzzy inference system for microservice resource management to achieve the best resource expansion strategy for various microservices in an edge computing network.
(4) To verify the effectiveness and performance of the proposed platform and algorithm, we perform extensive experiments in six scenarios of the Internet of Vehicles edge computing network. The experimental results show that the proposed approach improves microservice resource management efficiency in terms of reaction time and reduces surge burdens.

The remainder of this paper is organized as follows. Section 2 discusses the research background and the related literature. Section 3 presents the system model and proposed FMCRS. Section 4 describes the system architecture, microservice resource management platform, and system implementation in detail. Section 5 evaluates the proposed platform and algorithm through extensive experiments. Section 6 concludes the study and proposes some suggestions for future work.

## 2. Background and Related Work

In this section, we first introduce the research background for the issues to be addressed in this paper. Subsequently, we review and discuss two major approaches in the related literature, namely, system architecture/framework and management mechanism/algorithm.

The resource management of microservices has always been an important research topic in academia and industries [27,28,29,30]. Taherizadeh and Stankovski [31] presented a survey of the taxonomy and challenges related to auto-scaling applications in edge computing. They investigated various types of edge computing applications and their auto-scaling challenges when dynamic workloads occur. The authors discussed nine major technologies related to the auto-scaling application of edge computing: the cloud framework, virtualization technology, monitoring approach, operational behavior, adjustment capability, architecture support, adaptation interval, scalability technique, and image delivery. Their work identified several research challenges that should be considered and addressed to enhance the performance of applications in edge computing. Qu et al. [32] investigated various microservice deployment strategies on the edge computing platform. They implemented microservices with Docker containers and conducted comprehensive experiments on four cases to explore the performance of microservice deployment strategies. Their experimental results showed that the computing resources of microservices in an edge computing network, such as CPU and memory, will vary dramatically as the service scenario changes. Deploying microservices in edge computing networks will face the problems of operating and managing microservices. Therefore, edge computing networks require flexible and diverse microservice management methods to cope with complex network environments.

Designing new network architectures and system frameworks to improve edge computing microservice resource management efficiency is an important research approach. Gand et al. [33] proposed a serverless architecture for cluster container applications in edge computing. They designed and implemented a traffic management scheme as a proof of concept to improve the performance of deploying microservices across clusters. They also implemented their serverless architecture and evaluated the performance of the architecture in terms of the memory and CPU usages of clusters, message round-trip time, and function invocation time. The simulation results showed that their proposed architecture exhibits advantages, including reusability, scalability, and interoperability. In [34], Taherizadeh et al. proposed a capillary computing architecture for dynamically orchestrating microservices from edge devices to fog and cloud service providers. Their architecture consists of an edge/fog/cloud monitoring system and a capillary container orchestrator. The authors implemented all the necessary microservices as Docker containers and used a car equipped with a special communication hardware system as an edge computing node that could connect to a fog and cloud computing server. The experiment results showed that their proposed capillary computing architecture achieved a high quality of service (QoS) with a faster service response time. Alam et al. [35] developed a modular and scalable computing architecture based on lightweight virtualization. They combined Docker orchestration with a distributed deployment system to provide modularity. Their proposed architecture was able to minimize the effect of the failure of devices and microservices by quickly masking application logic across different layers. They tested the proposed architecture through experiments, and the results showed that it exhibited advantages such as fault tolerance and microservice deployment availability across different system architecture layers.

Yan et al. [36] presented a 5G satellite edge computing framework based on the microservice architecture to reduce delays and expand network coverage. Their framework consisted of edge computing microservices and embedded hardware platforms in satellites. All the computing resource nodes were virtualized into a resource management platform for deploying microservices in the framework. The authors performed a series of experiments to validate their proposed framework, which exhibited broad network coverage, as well as less delay and a lower packet loss rate than the ground 5G network. Forestiero, et al. [37] presented a novel ant-inspired self-organizing framework for service discovery and composition with an evolutionary-based approach, which can broadly be used in the Internet of Things and edge computing network. They proposed an algorithm for reorganizing and discovering service descriptors. Their algorithm can intelligently collect and reorganize service discovery requests to achieve the discovery of multiple basics services with a single query. The authors verified its performance in terms of the capacity of reorganizing service descriptor keys and service discovery operations via extensive simulation experiments. The experiment results showed that the designed framework and algorithm was indeed able to effectively reduce the number of service explorations in the Internet of Things and edge computing networks, shortening the time of the service search and reducing the network load.

Some researchers have attempted to design various novel mechanisms and policies to tackle the issues related to managing microservice computing resources in edge computing. Cicconetti et al. [38] designed two mechanisms for dynamically allocating microservices in edge computing networks at short timescales. They adopted the multi-access edge computing (MEC) standard of the European Telecommunications Standards Institute (ETSI) and implemented their proposals with open-source tools. The authors compared three microservice allocating operation approaches, namely, static, centralized, and distributed assignments, through experimental evaluations. They pointed out that the auto-scaling mechanism performs well when it has a systemic view of the usages of all the MEC hosts and demands. The authors also indicated that proposing a solution that can fit all the conditions in serverless edge computing is extremely difficult. Pallewatta et al. [39] designed a decentralized microservice placement policy for heterogeneous and resource-constrained fog computing environments. In their proposed policy, microservices are placed as close as possible to the data source to minimize latency and network usage. The authors also attempted to address the challenges of microservices related to service discovery and load balancing. They evaluated their policy through simulations, and the results showed that their proposed microservice placement policy achieved an 85% improvement in network latency and usage compared with the cloud-only placement approach.

The deployment of containerized microservices in edge computing has become increasingly important in recent years. Most current studies have focused on designing new system architectures or new microservice deployment methods. To date, however, discussions on providing efficient solutions to the service resource management of microservices in edge computing remain minimal. The current work presents an innovative prototype of an edge computing network microservice resource management platform. In contrast with the aforementioned studies, we designed and implemented a microservice resource management fuzzy inference system based on fuzzy theory and PSO.

## 3. System Models 

This section consists of two parts: the description of the model and the content of the FMCRS algorithm. First, we define the membership functions and calculation methods of the fuzzy management system for edge computing microservice resource management. Then, we separately describe the four core components of FMCRS, including the generation of fuzzy sets and rules, the optimization of fuzzy system membership functions, horizontal scaling, and vertical scaling mechanisms.

### 3.1. Description of Models 

We assume that the resource status data of microservices running on an edge computing network are *n* pairs of data. Each pair of data includes a *z*-dimensional input and a *k*-dimensional output. If the number of rules of the resource fuzzy inference system of a microservice is *m*, then a certain rule *l* in the fuzzy inference system can be expressed as Equation (1) [40]:

(1) $$\begin{array}{c} \mathrm{If}\;x^{1}\;is\;H_{l}^{1}\;\mathrm{and}\;\ldots \;\mathrm{and}\;x^{i}\;is\;H_{l}^{i},\\ \mathrm{then}\;y_{1}\;is\;M_{1}^{l}\;\mathrm{and}\;\ldots \;\mathrm{and}\;y_{j}\;is\;M_{j}^{l}; \end{array}$$

where $H_{l}^{i},\;i\in \left\{1,\;\ldots ,\;z\right\}$ and $M_{i}^{l},\;j\in \left\{1,\;\ldots ,\;k\right\}$ are fuzzy sets that define the input and output of our microservice fuzzy resource management inference system. $H_{l}^{i}$ designates the computing resource information related to microservices running on edge computing nodes, e.g., CPU and memory usages. $M_{i}^{l}$ signifies the extent of microservice resource scaling. We assume that the shapes of the membership functions of fuzzy sets $H_{l}^{i},\;i\in \left\{1,\;\ldots ,\;z\right\}$ and $M_{i}^{l},\;j\in \left\{1,\;\ldots ,\;k\right\}$ are Gaussian. Then, the Gaussian membership function of the microservice resource fuzzy sets can be defined according to Equation (2) [41]:

(2) $$\mu_{M_{J}^{l}}(x)=\exp\left[-\frac{1}{2}{\left(\frac{x-\mu_{l}}{\sigma_{i}^{2}}\right)}^{2}\right]$$

where $\mu_{l}$ and $\sigma_{i}$ denote the center and width of the *l*th fuzzy set $M_{i}^{l}$, respectively. If the fuzzy set of the CPU loading of a microservice is $\tilde{A}$ and the fuzzy set of its memory loading is $\tilde{B}$, then the membership function of the microservice resource management fuzzy inference system is calculated using Equation (3):

(3) $$\theta =\mu_{\tilde{A}\cap \tilde{B}}(x)=\wedge \left[\mu_{\tilde{A}},\mu_{\tilde{B}}\right]=\min\left[\mu_{\tilde{A}},\mu_{\tilde{B}}\right]$$

This fuzzy system integrates all the rules and defuzzifies them with a weighted average method to obtain a clear fuzzy system output value θ, as shown in Equation (4), where $w_{i}$ is the fitness of each rule.

(4) $$\theta =\frac{\sum_{i=1}^{n}w_{i}\times \theta_{i}}{\sum_{i=1}^{n}w_{i}},\;\mathrm{where}\;w_{i}=\mu_{{\tilde{A}}_{i}}(x)\wedge \mu_{{\tilde{B}}_{i}}(x)$$

### 3.2. FMCRS Algorithm

In this study, we design the FMCRS algorithm on the basis of fuzzy theory. The FMCRS algorithm consists of four major steps: (1) generating fuzzy sets and rules, (2) optimizing the fuzzy membership function, (3) horizontal scaling, and (4) vertical scaling.

#### 3.2.1. Generation of Fuzzy Sets and Rules

Through some open-source tools, network users can easily detect and collect the computing resource usage data of each microservice running on an edge network’s computing nodes. We use CPU and memory usages as the input of fuzzy sets. The use of CPU and memory by microservices is divided into three levels: high, medium, and low. The scaling extent of the computing resources of microservices is the output of the fuzzy inference system. We define the scaling extent as the amount of computing resource capacity that needs to be properly expanded when the computing resources of a certain microservice are insufficient in the edge computing network environment. The scaling extent is a percentile measurement. In the fuzzy inference system, the scaling extent is converted into a format that conforms to the definition of the fuzzy rules, and it is converted into three levels—high, medium, and low—according to its percentage value. The scaling extent is calculated using the FMCRS algorithm designed in this article. Therefore, the designed fuzzy sets include two input parameters, one output parameter, and seven fuzzy rules. Table 1 provides the fuzzy rules for the proposed microservice resource scaling management.

**Figure 1:**
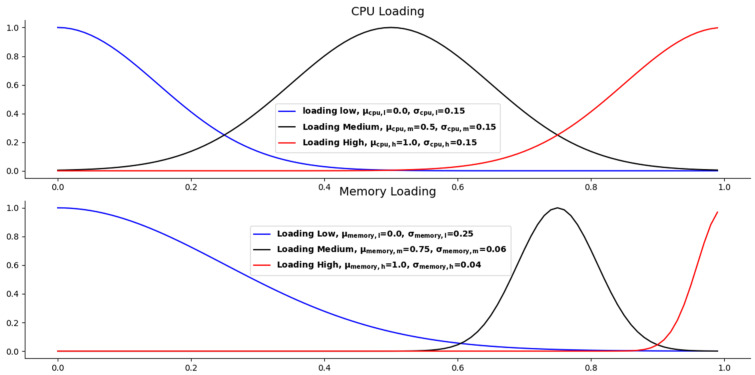

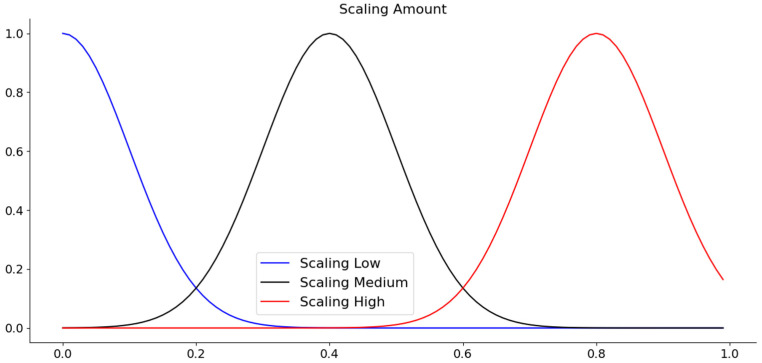
Gaussian membership function of the microservice resource management fuzzy sets.

Figure 1 presents the Gaussian membership functions of our microservice resource management fuzzy sets. The Gaussian membership function is often used to represent vague and linguistic expressions, such as “increases” or “increases little”. The Gaussian membership function of the microservice resource fuzzy sets is given in Equation (2), where $\mu_{l}$ and $\sigma_{i}$ are the center and width of the *l*th fuzzy set, respectively. Membership values are computed for each input value in x. The subgraph in the upper part of Figure 1 presents the distribution of the Gaussian membership values of the microservice CPU loading. This subfigure shows the distribution of the Gaussian membership values when the CPU loading of the microservice is high, medium, and low, respectively. The middle subgraph in Figure 1 shows the distribution of the corresponding Gaussian membership values when the loading of the microservice memory is high, medium, and low. The lower subgraph in Figure 1 shows the three distributions of Gaussian computing resources scaling extent when the CPU and memory loadings of the microservice are high, medium, and low respectively.

**Figure 2:**
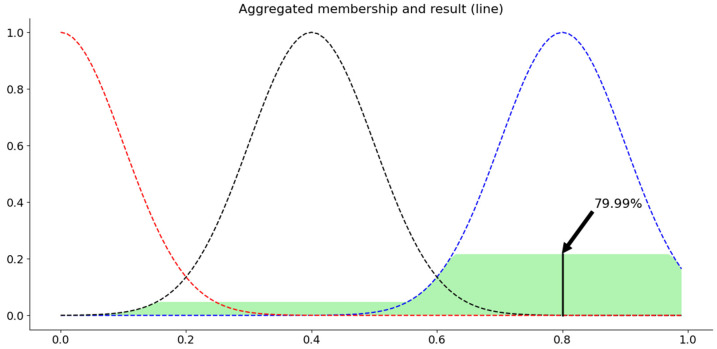
Use case of the microservice resource management fuzzy interference system.

Figure 2 shows a use case of our fuzzy inference system for microservice resource management. When the CPU loading of a microservice is 87% and the memory loading is 93%, our fuzzy inference system estimates the resource scaling extent of this microservice to be 79.99%.

#### 3.2.2. Optimization of the Fuzzy Membership Function

PSO is an algorithm developed by Kennedy and Eberhart [42] to observe the foraging behavior of birds. In the PSO algorithm, a particle represents an individual in a flock of birds. Each particle has a “memory” and refers to the “messages” of other particles to determine the direction of its movement. The behavior of a single particle may be assumed as unpredictable, but each particle refers to the information of other particles, and thus the behavior of the group is actually predictable. This condition is attributed to particles which correct the direction of their movement after determining that other particles are better than them, and thus refer to these particles and learn from others. Therefore, we can assume that all particles will gradually move in a better direction. The PSO algorithm can be used to solve the optimization problem. The mathematical expression of the PSO algorithm is provided in Equation (5):

(5) $$\begin{array}{l} v_{i}^{t+1}=\tau \times v_{i}^{t}+c_{1}\times rand()\times (p_{i}-x_{i}^{t})\\ \;\;+c_{2}\times rand()\times (g-x_{i}^{t}) \end{array}$$

(6) $$x_{i}^{t+1}=x_{i}^{t}+v_{i}^{t+1}$$

where $v_{i}^{t+1}$ is the velocity of the *i*th particle at time *t+1*, and $x_{i}^{t}$ is the position of the *i*th particle at time *t*. $p_{i}$ is the best position that the *i*th particle has ever traveled. *g* is the best position that all the particles have ever traveled. $c_{1}$ is the weight of individual experience, and $c_{2}$ is the weight of group experience. The calculation method for the latest position of particle *i* at time *t+1* is provided in Equation (6). The PSO algorithm uses a fitness function to determine where the particles should go, i.e., the best position, being the position with the most food sources for birds.

The membership function of the fuzzy inference system considerably influences the performance of the system; thus, selecting the best membership function, including the shape of the function, the number of fuzzy rules, and the parameters of the function, affects the accuracy of the fuzzy inference system. Accordingly, the current study uses the PSO algorithm to solve the problem of optimizing the designed fuzzy inference system of microservice resource management. Let $\mu_{i}$ and $\sigma_{i}$ be the center value and standard deviation of the fuzzy variables of the Gaussian membership function. We define a mean squared error (MSE) fitness function to evaluate the performance of our fuzzy inference system, as shown in Equation (7), where $S_{i}$ is the real amount of computing resources scaled by a microservice, and ${\hat{S}}_{i}$ is the estimated amount of computing resources that a microservice needs to scale. When MSE is smaller, the performance of the fuzzy inference system is better. Therefore, the task of using the PSO algorithm to optimize the fuzzy inference system for microservice resource management involves using the fitness function as Equation (7) and continuously and recursively guiding the optimization of the $\mu_{i}$ and $\sigma_{i}$ parameters to achieve the best system performance.

(7) $$MSE=\frac{\sum_{i=1}^{n}{\left(S_{i}-{\hat{S}}_{i}\right)}^{2}}{\sum_{i=1}^{n}S_{i}^{2}}$$

#### 3.2.3. Horizontal Scaling

The horizontal scaling mechanism of the FMCRS algorithm can create new microservices on the same edge computing node without having to migrate microservices that requiring resource expansion to new edge computing nodes. When a microservice on an edge computing node needs to expand computing resources, the FMCRS will first check whether the amount of computing resources required by the microservice is lower than the computing resources available on the edge computing node where the microservice is located. If it is lower than the computing resources available to the computing node, the FMCRS will start the horizontal scaling mechanism, and directly allocate the required computing resources to the microservice from the computing node where the microservice is currently located. This mechanism ensures that the microservice application can continue to execute, and avoids the short interruption caused by the microservice being migrated to other nodes.

**Figure 3:**
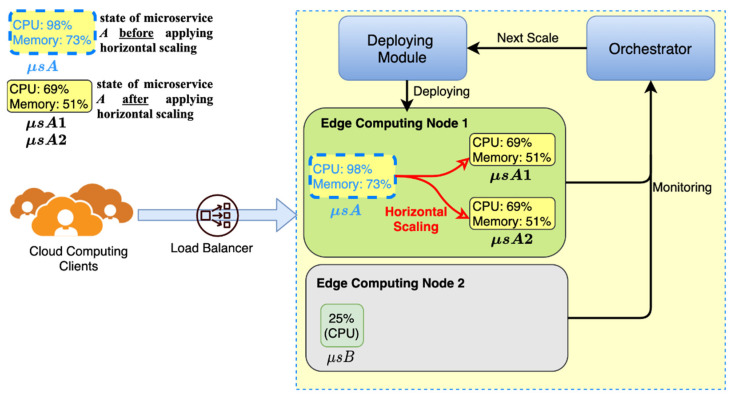
Horizontal scaling of the FMCRS algorithm.

Figure 3 presents an example of the horizontal scaling mechanism. The dotted line microservice in the figure is the state before the operation, and the solid line microservice is the state after operation. In Figure 3, edge computing nodes 1 and 2 run microservices A and B, respectively. The red solid line in the figure indicates that the horizontal scaling mechanism of the FMCRS algorithm has expanded the computing resources of microservice A. The solid red line connects the two operating states of microservice A, indicating that the state of microservice A has changed. The dotted subgraph represents the state before the horizontal scaling of the computing resource is applied, and the solid line subgraph represents the state after the horizontal scaling mechanism of the computing resource is applied. When microservice A requires resource expansion, and the microservice resource management fuzzy inference system estimates that the resource expansion of microservice A is 40% and the available computing resources of node 1 are sufficient to meet the resource expansion demand of microservice A, then FMCRS expands horizontally, creating two copies of new microservice A on node 1, i.e., microservices A1 and A2, and allocating new computing resources (CPU: 69%, memory: 51%) for the two new microservices. The orchestrator is the resource manager of the fuzzy inference system, designed for microservice resource management. We describe the implementation methods of each component of the system in detail in Section 4.

#### 3.2.4. Vertical Scaling

Compared with those of cloud computing networks, the computing resources of edge computing networks are limited, and an increase in hardware computing resources is inelastic. Differently from the horizontal scaling mechanism, vertical scaling will migrate microservices that require the expansion of computing resources to new edge computing nodes. When a microservice on an edge computing node needs to expand the computing resources, if FMCSR estimates that the required expansion resources are greater than the remaining available computing resources on the edge computing node where the microservice is located, the microservice uses the vertical scaling method to perform the resource expansion. Vertical scaling can reasonably distribute the load of each edge computing node.

An application composed of microservices has different priorities at each edge computing node. Thus, when many microservices are required to be migrated to other edge computing nodes, FMCRS must still determine which microservice should be moved to the node with a lower resource load based on the priority of each microservice. Equation (8) is used to define the priority of the adjustment resources of each microservice. $cpu_{\mu s}$ and $memory_{\mu s}$ denote the CPU and memory computing resources occupied by microservices. $cpu_{node}$ and $memory_{node}$ represent the CPU and memory usage ratios of edge computing nodes. In practical applications, the priority of microservices can be flexibly defined in accordance with the needs of users. For example, priority can be defined on the basis of the purpose and importance of a microservice. FMCRS migrates microservices with lower priority to other computing nodes with a lower load to ensure that microservices with higher priority have sufficient resources to continue running on the original computing nodes. After FMCRS creates new microservices on new nodes, it releases the computing resources originally occupied by the microservices with lower priority.

(8) $$Priority_{\mu s}=\frac{cpu_{\mu s}\times memory_{\mu s}}{cpu_{node}\times memory_{node}}$$

**Figure 4:**
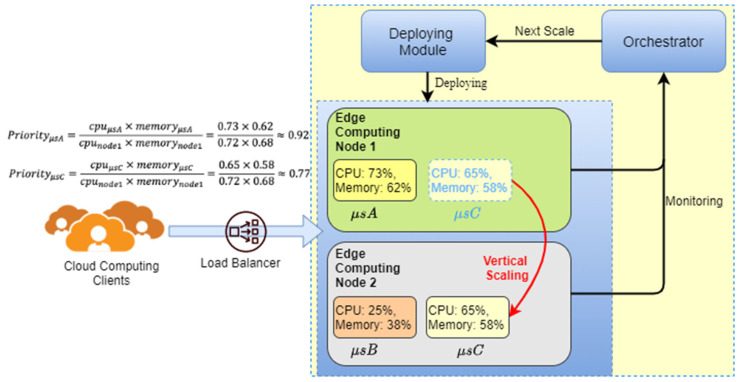
Vertical scaling of the FMCRS algorithm.

Figure 4 is an example of the vertical scaling mechanism of FMCRS. There are two edge computing nodes in the figure. Among them, two microservices, A and C, run on computing node 1. Microservice B runs on computing node 2. When microservice C needs to expand computing resources, because the available computing resources of node 1 are insufficient to meet the resource expansion required by microservice C, this situation will activate the FMCRS vertical scaling mechanism, as illustrated by the red solid line in Figure 4. As microservice C has a lower priority than microservice A, FMCRS migrates microservice C from node 1 to node 2, and at the same time releases the computing resources occupied by microservice C in node 1.

#### 3.2.5. Algorithm Pseudocode

FMCRS is summarized in Algorithm 1. First, we initialize the fuzzy sets and parameters of the microservice resource computing fuzzy management inference system. When an iteration occurs, the algorithm calculates the fuzzy membership functions of all the fuzzy sets for each microservice on all the edge network’s computing nodes (Steps 1 to 3). Then, FMCRS optimizes the fuzzy membership functions using the PSO algorithm (Step 4). All the fuzzy rules are integrated and defuzzified to calculate the estimated required scaling computing resources: $s_{r_{cpu}}$ and $s_{r_{mem}}$. If the required resources of a microservice are less than the computing resources available to the computing node where it is located, then horizontal scaling is implemented. Otherwise, vertical scaling is performed in accordance with the priority of the microservice (Steps 5 to 13). If no edge computing nodes for vertical resource scaling are available, then microservices with lower priority are terminated to ensure that microservices with higher priority can continue to operate normally.
**Algorithm 1** Algorithm for fuzzy-based microservice computing resource scaling (FMCRS)**Initialization:**
$\mathrm{Edge}\;\mathrm{computing}\;\mathrm{nodes}\;\mathrm{set}\text{:}\;M_{i}=\left\{1,\;2,\;3,\;\ldots ,\;M\right\},\;0<i\leq M$; $\mathrm{CPU}\;\mathrm{usage}\;\mathrm{for}\;\mathrm{edge}\;\mathrm{node}\;\mathrm{set}\text{:}\;M_{i_{cpu}},\;i\in M$; $\mathrm{Memory}\;\mathrm{usage}\;\mathrm{for}\;\mathrm{edge}\;\mathrm{node}\;\mathrm{set}\text{:}\;M_{i_{mem}},\;i\in M$; $\mathrm{Microservices}\;\mathrm{set}\text{:}\;N_{i}=\left\{1,\;2,\;3,\;\ldots ,\;N\right\},\;0<i\leq N$; $\mathrm{CPU}\;\mathrm{usage}\;\mathrm{for}\;\mathrm{microservice}\;\mathrm{set}\text{:}\;K_{i_{cpu}},\;i\in N;$ $\mathrm{Memory}\;\mathrm{usage}\;\mathrm{for}\;\mathrm{microservice}\;\mathrm{set}\text{:}\;K_{i_{mem}},\;i\in N;$ Gaussian membership function of fuzzy interference system with standard deviation *σ* and mean value *μ;* $\mathrm{microservice}\;\mathrm{scaled}\;\mathrm{CPU}\;\mathrm{extent}\;\mathrm{set}\text{:}\;S_{r_{cpu}}=\emptyset ,\;r\in N$; $\mathrm{microservice}\;\mathrm{scaled}\;\mathrm{Memory}\;\mathrm{extent}\;\mathrm{set}\text{:}\;S_{r_{mem}}=\emptyset ,\;r\in N$.1:**For** Each microservice i∈N**Do**2:**For**$\;\mathrm{each}\;K_{i_{cpu}}$$\;\mathrm{and}\;K_{i_{mem}}$**Do**3:**  **Calculate Gaussian membership values according to (2) and (3), respectively;4:**  **Optimize Gaussian membership function according to (5), (6), and (7), respectively;5:**  **$\mathrm{Calculate}\;S_{r_{cpu}}$$\;\mathrm{and}\;S_{r_{mem}}$ according to (4);6:**  if**$S_{r_{cpu}}<\left(1-M_{i_{cpu}}\right)$ $\mathrm{and}\;S_{r_{mem}}<\left(1-M_{i_{mem}}\right)$ **then**7:**    **Run horizontal scaling;8:**  else**9:**    **Calculate the priority of microservice according to (8);10:**    **Run vertical scaling;11:**  end if**12:** end for**13:**end for****Output:** Microservices with the scaled computing resources.

## 4. System Architecture and Implementation

This section presents the system architecture design and implementation in detail. We describe the conceptual model and representation of the system with various diagrams. We also show how our proposed platform is physically built and operated.

### 4.1. System Architecture and Design

**Figure 5:**
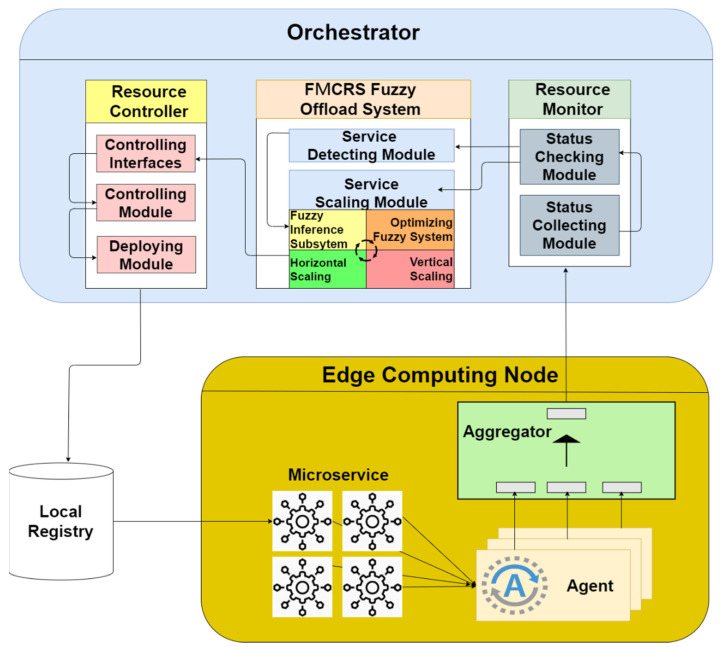
The system architecture of the fuzzy-based microservice resource management platform for edge computing.

We designed and implemented the edge computing microservice management platform prototype from the perspective of the network operator. The platform can provide network service operators with multiple microservice resource management mechanisms to achieve the scalability of computing resources. The platform includes a microservice computing resource fuzzy off-loading system. This system can implement functions, such as resource monitoring and deployment management, under different microservice resource usage conditions. The platform architecture is illustrated in Figure 5.

Our platform refers to the architectures proposed in the MEC architectural documents [43,44,45] published by ETSI. We improve the function of resource control by using lightweight microservice technology and orchestration agent architecture to reduce the burden of system management. Our platform uses microservices to deploy applications that can solve the problem of sensitive edge network delay time. Given the independence and high flexibility of microservices, they can still be replaced with other microservices of the same nature when a service failure occurs or a software version update is required.

In the designed platform system architecture, the orchestrator is responsible for the centralized resource management of the entire edge computing network environment. It provides the most basic centralized operations for each microservice, including resource allocation, service deployment, and microservice status adjustment. The resource monitor module contains a variety of microservice operating status detection programs to collect the operating statuses of microservices, including connection, network delay, and computing resource occupancy statuses. When the cloud computing network deploys microservices to the edge computing nodes in accordance with the client’s requirements, the resource monitor on the platform monitors the status of each edge computing node in real-time.

The FMCRS offload system module is responsible for the allocation and management of the computing resources of each microservice. The designed algorithm is implemented in this module. This algorithm adjusts the edge network on the basis of the collected data and triggers the execution control module to issue resource adjustment rules and operating instructions to each edge computing node. The lower layer in the platform architecture diagram shows the collection of all the edge network’s computing nodes. Each edge computing node runs the microservices required by the cloud computing network. Here, each microservice has an “agent” responsible for monitoring and receiving data, and then the aggregator deployed on each edge computing node periodically collects the status of each microservice and sends it back to the upper data collector. Microservices use a RESTful API [46] to communicate autonomously with other microservices.

### 4.2. System Implementation

To verify the effectiveness of our proposed microservice resource management platform prototype for edge computing, the designed management platform was implemented on Xen servers. Table 2 and Table 3 provide the hardware and software specifications of the proposed fuzzy-based microservice resource management platform. We implemented our system with Python and Java programming language.

We implemented two resource management mechanisms in the designed edge computing microservice resource management platform prototype. The purpose was to realize the resource utilization of microservices and reduce resource consumption. For example, the resource detection and scaling mechanisms in the platform aim to detect the load situation of the computing resources of microservices in time and realize the automatic expansion function of computing resources. The purpose of the resource adjustment mechanism is to reduce the consumption of microservice computing resources and improve the rate of obtaining microservice resources. The aforementioned multiple mechanisms, combined with the FMCRS algorithm, achieve a high degree of autonomy in system and microservice management. The implementation methods of each system module and management mechanism are described in detail in the succeeding sections.

The control module in the resource controller component implements common management functions for microservices, including deployment, scaling, deletion, and configuration. The control module deploys a microservice with specifications, including whether the type of microservice is single or expandable. The deployment specifications also include basic initialization setting instructions and microservice external network communication ports. Moreover, this module is responsible for providing configuration files that should be downloaded and executed when a microservice is started. All the configuration files are stored in the form of YAML scripts. A microservice downloads and executes these configuration files for normal operation.

**Figure 6:**
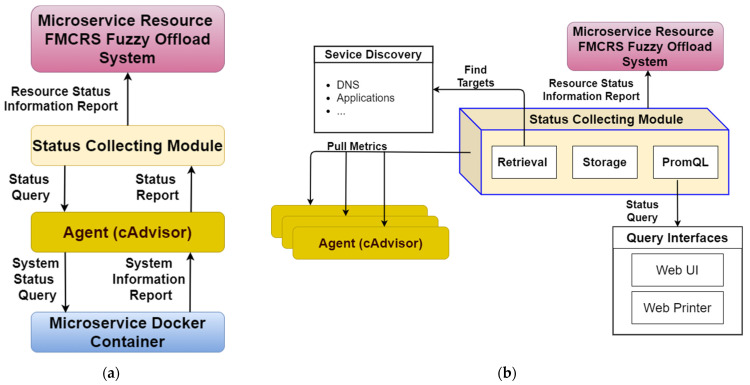
Components of the status-collecting module and the associated external components. (**a**) Flowchart of information exchange between the status-collecting module and cAdvisor; (**b**) diagram of the internal components of the status-collecting module.

The status-collecting module is responsible for collecting and storing data related to microservices. After the resource controller deploys a microservice, this controller transmits the status data of the microservice to the status-collecting module through an agent. cAdvisor can be used as the collection agent for the microservice status data. It accesses container-based microservices and resources on each node, including CPU usage and memory usage. In the designed status-collecting module, Prometheus is used to access each cAdvisor actively. Figure 6 shows the components of the status-collecting module and the associated external components. Figure 6a presents the flowchart of information exchange between the status-collecting module and cAdvisor. Figure 6b depicts a diagram of the internal components of the status-collecting module. As shown in Figure 6b, the status-collecting module includes three functional components: (1) retrieval is responsible for capturing data from the target, (2) storage is responsible for accessing data, and (3) PromQL provides the Prometheus query language component. The status-collecting module is also built on each edge computing node in the form of microservices, and it actively collects computing resource measurement data from each node and regularly records data.

We implemented the designed FMCRS algorithm in the FMCRS fuzzy offload system and requested the resource controller to adjust the corresponding resources of the computing nodes in the edge computing network. The FMCRS algorithm has four important computing mechanisms: a fuzzy inference system, optimization of the fuzzy system, horizontal scaling, and vertical scaling. These four computing mechanisms are implemented in the service detecting and scaling modules. These modules periodically read the status-collecting module data to obtain the latest status of microservices running on each edge computing node. Then, the FMCRS algorithm is automatically executed in accordance with the status of the microservices to adjust the resource usage requirements of the microservices and achieve the efficient use of resources.

The status-collecting module receives the service functions and status data of each microservice every second and then sends these status data to the service-detecting module. The latter analyzes the CPU and memory usages of microservices to calculate the probability of expanding their computing resources. The calculation method for the CPU and memory usages of the microservice is provided in Equation (9), where $cpu_{\mu s}(y)$ and $memory_{\mu s}(y)$ denotes the CPU usage and memory usage of microservices on the edge computing nodes. $cpu_{node}(x)$ and $memory_{node}(x)$ are the available CPU and memory usages of the edge computing nodes.

The service scaling module dynamically adjusts the number of microservices based on user needs and the calculation results of the service detection module to maintain the quality of service of the computing system and reduce the deployment cost of network service providers. This module calculates the number of additional microservices that are required to be deployed on the basis of the resource usage of microservices in real-time and then sends the results to the deployment module in the resource controller component to filter new edge computing nodes for the corresponding microservice deployment. When the external cloud network accesses the computing nodes in the edge network, the load balancer delivers the computing services to the corresponding microservices.

(9) $$\begin{array}{c} CPU_{usage}=\frac{cpu_{\mu s}(y)}{cpu_{node}(x)},\\ Memory_{usage}=\frac{memory_{\mu s}(y)}{memory_{node}(x)},\\ \mathrm{where}\;0<x\leq node\;quantity,\;\mathrm{and}\;0<y\leq microservice\;quantity. \end{array}$$

## 5. System Evaluation and Results

**Figure 7:**
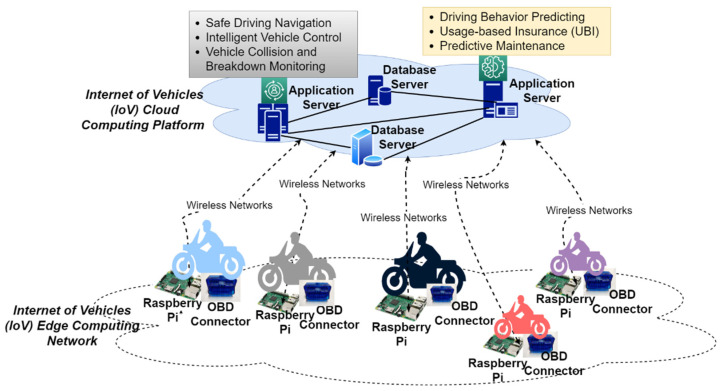
Experiment evaluation system architecture.

To verify that the platform we designed can be effectively applied in the edge computing environment of the Internet of Things, we examined the use case of Internet of Vehicles (IoV) cloud computing, with edge computing as the experimental performance analysis environment. The experimental system architecture diagram is shown in Figure 7. The IoV cloud computing platform consists of various database servers and application servers. These application servers provide various IoV application services for connected vehicles, such as driving behavior analysis and prediction, predictive maintenance, safe driving navigation, UBI insurance, etc. The IoV edge computing network comprises multiple connected motorcycles. Each motorcycle is an edge computing node implemented with an on-board diagnostics (OBD) connector and a Raspberry Pi minicomputer. OBD connectors collect vehicle condition data and driving behavior data. There are more than 30 types of data collected, including vehicle speed, driving distance, engine revolutions per minute (RPM), fuel consumption, intake air temperature, etc. The OBD connector transfers the collected data to the Raspberry Pi mini-computer via Bluetooth. The Raspberry Pi mini-computer then transmits the collected data to the IoV cloud platform via the wireless network.

The collected data volume is extremely large since the OBD connectors collect vehicle condition data and driving behavior data per second. To reduce the amount of data sent to the cloud platform, reduce the cost of cloud computing, and reduce the delays in application services, we performed edge computing on the Raspberry Pi mini-computer installed on the motorcycle. We deployed some application microservices of the cloud computing platform on the raspberry pi mini-computer of the motorcycle. These microservices can perform filtering, cleaning, and preprocessing of big data, and then send the preprocessed results to the IoV cloud computing platform. Six experiments were conducted on the basis of the implemented microservice resource management platform in such an IoV cloud/edge computing network, and the performance of FMCRS was compared with a state-of-the-art microservice scaling management method.

We conducted a series of experiments in six scenarios to examine the performance of our proposed microservice resource management method from three aspects. Experiments 1 to 3 aimed to verify that some necessary microservice computing resource management functionalities were able to be realized on the platform we designed. Experiments 4 and 5 were designed to test the performance of the proposed horizontal and vertical scaling mechanism. Experiment 6 compared the performance of the FMCRS mechanism and the Kubernetes auto-scaling mechanism in the stress test.

### 5.1. Experiment 1: Microservice Deployment Time on the Designed Platform

This experiment was used to test the time required to deploy different types of microservices on the designed platform. We divided the necessary services of IoV applications into various types of microservices and then deployed them on each computing node in the edge computing network to test the deployment time of these microservices. The microservices have 11 types, i.e., three types of database microservices and eight types of website core function microservices. These microservices are as follows: carts, carts-db, catalogue, front-end, pay, rabbit, queue, order-db, order, user-db, and the user.

**Figure 8:**
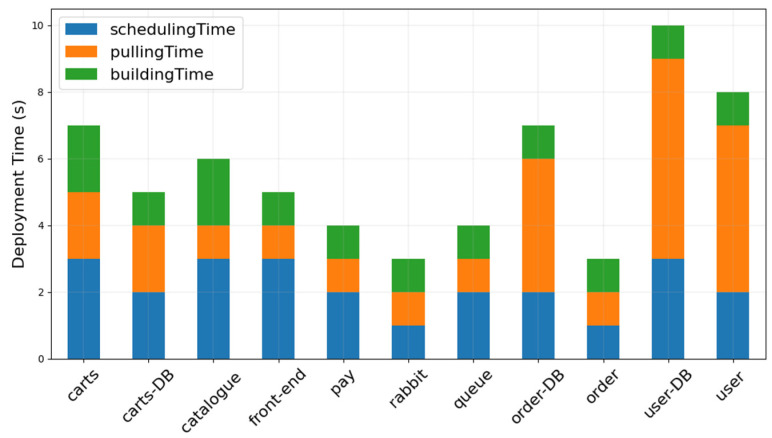
Microservice deployment time on the designed fuzzy-based microservice resource management platform.

The deployment of all the microservices consists of three steps: scheduling, pulling, and establishment. In the scheduling phase, the microservices are allocated to the corresponding computing nodes of the edge network in accordance with the deployment algorithm. The pulling phase pulls down the Docker images of various microservices from the Docker repository to each computing node. During the establishment phase, some basic functions and configurations are configured to the microservices. The first two of the three stages take a longer time because the microservice deployment algorithm in the scheduling phase analyzes the resource requirements of each microservice and the available resources of each computing node to determine the deployment location of a microservice. In the pulling phase, the size of the Docker image of a microservice affects the download time. The larger the Docker image of a microservice, the longer the pulling time. Compared with the first two stages, the establishment time of microservices is relatively stable because only the basic parameters of the microservice startup should be initialized, and thus, the establishment time of each microservice does not differ considerably. As shown in Figure 8, regardless of which microservice is deployed on the designed microservice resource management platform, the process can be completed within 10 s. Thus, our platform can quickly deploy various microservices in an edge computing network.

### 5.2. Experiment 2: Computing Resource Scaling Time of Microservices 

**Figure 9:**
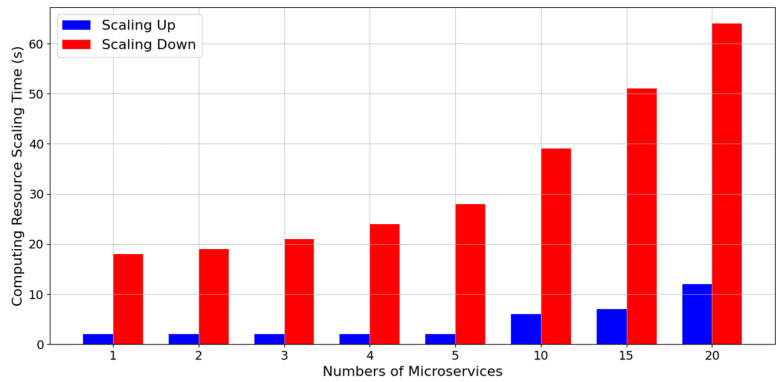
Computing resource scaling time of microservices on the designed fuzzy-based microservice resource management platform.

This experiment was used to test the time it takes to scale up and down different numbers of microservice computing resources on the designed platform. We tested the time required to scale up and down one to 20 microservices. As shown in Figure 9, given that we scaled up the resources of the microservices in a parallel manner, the time to expand two microservices was not two times but was extremely close to the time of expanding one microservice. In this manner, the time spent on scaling up microservice resources was considerably reduced.

When microservices require the scaling down of resources, the time required to reduce a microservice resource is not equal to the time required to expand a microservice. Moreover, it may even be four to five times the time required to scale up resources. Simultaneously scaling down the resources of 20 microservices takes more than 1 min because our system management platform does not forcefully delete microservices directly. Instead, the system adopts a sophisticated microservice termination process. In this process, microservices can be terminated and restarted at any time in a decentralized computing environment. When ending and restarting, however, microservices on edge computing nodes must first refuse to accept the service requirements of new users, and then microservices are terminated after the previously processed computing requirements are executed. Therefore, when microservices perform resource scaling down on our platform, they experience an exit time of a preset termination period.

The results of the experiment also proved that the time it took one microservice to scale up computing resources was 2.7 s on our platform. The time it took to scale up resources for 20 microservices simultaneously was 12.6 s. The scaling-down time of resources for one microservice was 18.1 s, and that for 20 microservices simultaneously was 62.3 s. The designed microservice resource management platform system displays the capability to process the scaling up and down of microservice computing resources rapidly in an IoV edge computing network environment with relatively insufficient computing resources.

### 5.3. Experiment 3: Computing Resource Monitoring of Edge Computing Nodes and Microservices

**Figure 10:**
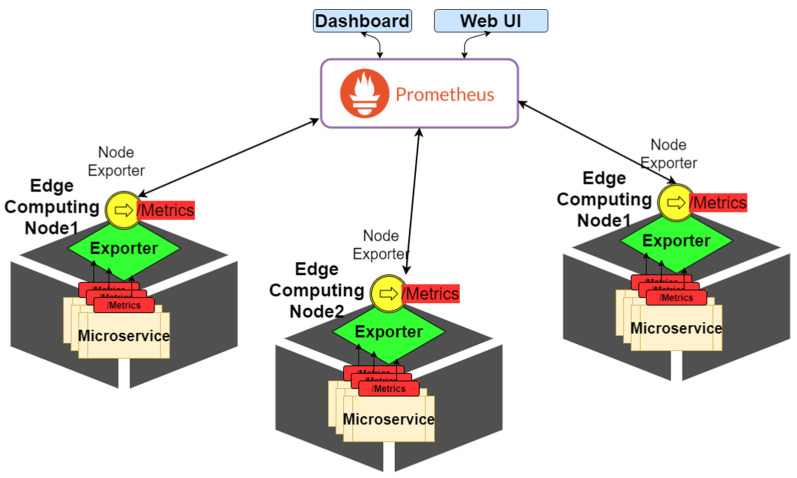
Computing resource monitoring architecture of the fuzzy-based microservice resource management platform.

This experiment aimed to verify that the designed platform can monitor the computing performance status of each computing node in an edge computing network and each microservice running on that node in real time. In this experiment, we used the open-source project Prometheus and deployed microservices on each edge computing node. We preinstalled an export program on each computing node, as shown in Figure 10. Through these export programs, the real-time collection of microservices on computing nodes and node resource performance data were stored in time in the created micro database. Therefore, the system administrator or user of our platform can obtain the latest computing resource performance status information by accessing only the database through the PromQL syntax. Our platform also uses the Grafana which is an open-source composable data visualization software package to deploy the collected computing resource performance status information on the webserver, enabling users to query it conveniently as a reference for microservice computing resource management.

**Figure 11:**
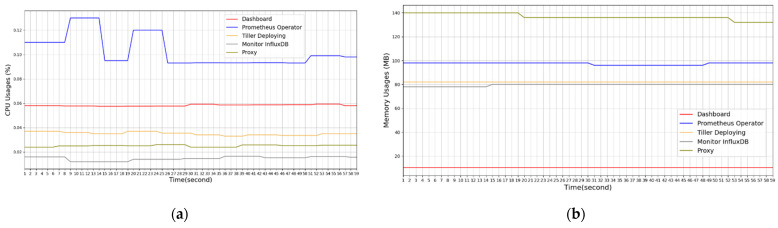
Computing usage of microservices monitored on the designed platform. (**a**) CPU usages of five microservices monitored. (**b**) Memory usages of five microservices monitored.

**Figure 12:**
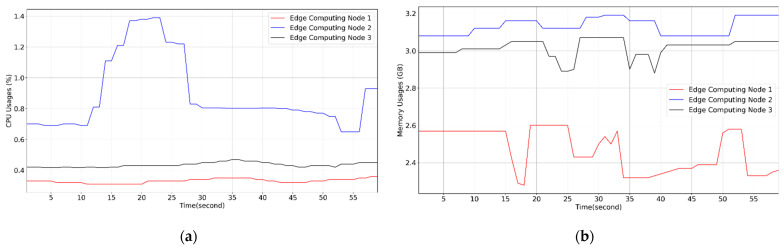
Computing usage of edge nodes monitored on the designed platform. (**a**) CPU usages of three edge computing nodes monitored. (**b**) Memory usages of three edge computing nodes monitored.

Figure 11 presents the results of the real-time monitoring of the CPU and memory resource statuses of the five microservices in this experiment. Figure 12 illustrates the results of the real-time monitoring of the CPU and memory resource statuses of the three computing nodes in the edge computing network in this experiment. The results of this experiment indicated that the designed platform can immediately detect the performance status of the computing resources of each computing node and microservice in the edge computing network, providing a basis for the dynamic resource management of the FMCRS algorithm.

### 5.4. Experiment 4: Horizontal Scaling of Microservices

This experiment was a continuation of the previous experiment to verify that the designed platform can quickly expand new microservices horizontally when it detects that microservices require resource expansion. The designed platform has a microservice detection mechanism. Thus, when it detects that the computing resource load of a certain microservice is too high and will trigger a resource expansion, the designed FMCRS algorithm can calculate the extent of resources that need to be expanded and starts a new microservice during the time when microservices off-load the computation of microservices that were originally overloaded.

**Figure 13:**
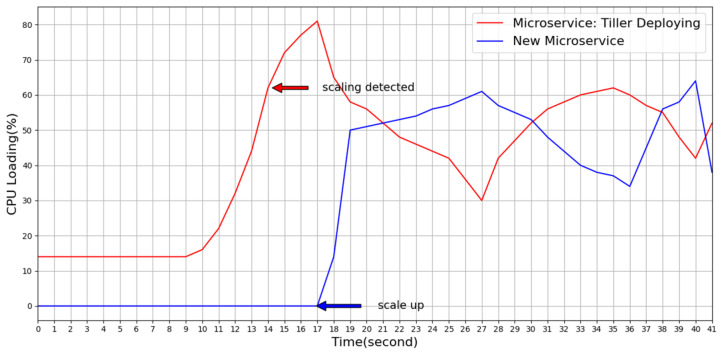
Horizontal scaling of a microservice on the designed platform.

As shown in Figure 13, our platform detected that the load of the microservice called “Tiller Deploying” was too high at the 14th second, and thus, it calculated the number of expanded microservices through the FMCRS algorithm. Thereafter, the FMCRS algorithm started a new microservice at the 17th second to share the computing traffic of microservices that originally required resource expansion. In accordance with the experimental results, the designed platform can thus detect the resource status of microservices in time and quickly expand the computing resources of microservices horizontally.

### 5.5. Experiment 5: Vertical Scaling of Microservices

This experiment verified that the designed FMCRS algorithm can vertically expand the computing resources of microservices. When the load of a computing node in an edge computing network is excessively high, the designed platform detects the abnormal state of the computing node’s resource usage in time and compares the priority of the computing node with the computing logic in our algorithm. Low microservices are removed from the computing node and migrated to other edge computing nodes with a lower load.

**Figure 14:**
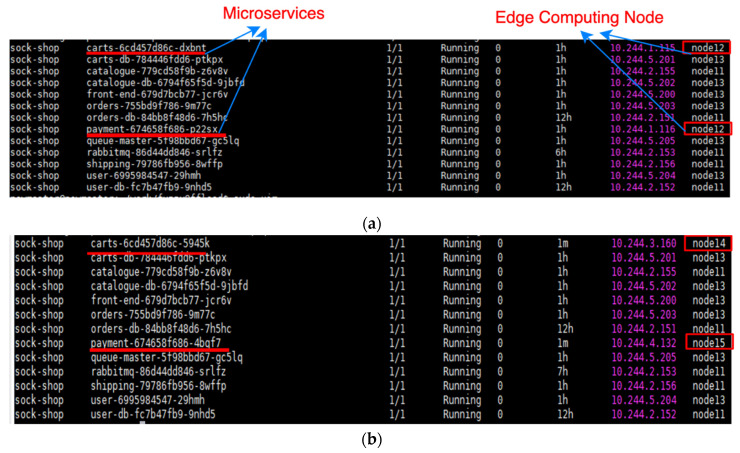
Vertical scaling of the FMCRS algorithm. (**a**) Microservice status before applying vertical scaling of the FMCRS algorithm. (**b**) Microservice status after applying vertical scaling of the FMCRS algorithm.

Figure 14 present the results of this experiment. In Figure 14a, the 11 microservices in Experiment 1 were originally running on computing node 12 of the edge network. However, given that the computing load of node 12 was excessively high, the FMCRS algorithm migrated two microservices (carts and payment) with lower priority to computing nodes 14 and 15, which had lower loads. Figure 14b illustrates the vertical scaling of microservice computing resources, i.e., the result of migrating to other computing nodes. Therefore, this experiment verified that the designed platform can effectively realize the rapid vertical scaling of microservice computing resources.

### 5.6. Experiment 6: Comparison of Microservice Computing Resource Scaling

**Figure 15:**
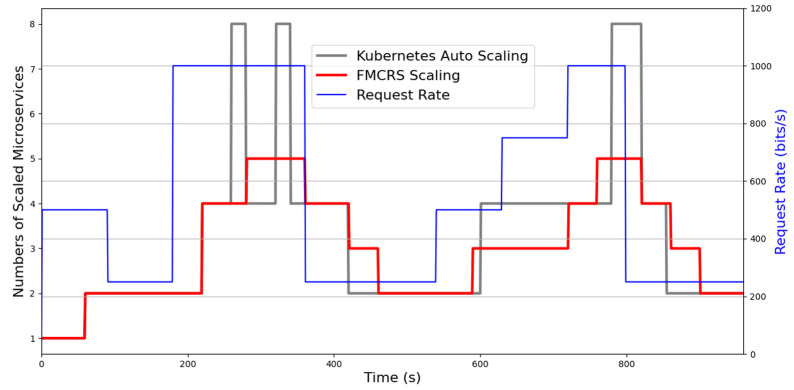
Comparison of the resource management of microservices between two scaling methods.

This experiment compared the efficiency of the designed FMCRS microservice resource management method with that of another cloud microservice resource management method, namely, Kubernetes auto-scaling. In this experiment, we applied JMeter which is an open-source software designed used to design load test functional behavior and measure performance to simulate a large number of edge node users accessing the cloud computing platform simultaneously. We used JMeter’s stepping thread group mode to send HTTP requests to the IoV application server. JMeter provides the interface of the dashboard to allow users to view the analysis results, such as network traffic. We implemented two resource management algorithms, namely, the designed FMCRS scaling and Kubernetes auto-scaling, on the microservice resource management platform and compared their performance. The parameters of the experimental environment are provided in Table 4. The initial number of microservices was one, and the maximum number of microservices deployed on the platform was 20. The total experiment time was 960 s, and JMeter’s service rate was updated every 180 s. In Figure 15, the X-axis is the experiment time, the Y-axis is the number of microservices, and the secondary axis of the Y-axis is the pressure of the IoV application server’s access demand, that is, the request rate (bits/s). The blue line shows the change in demand for simultaneous access services received by the servers over time.

We observed that the FMCRS scaling algorithm and the Kubernetes auto-scaling algorithm were able to reflect the demands for the expansion and reduction of microservice resources on the platform promptly. When the computing load of a microservice CPU or memory exceeded a high critical value, both algorithms were able to quickly expand microservice computing resources to achieve the load distribution. However, the resource expansion response of the Kubernetes auto-scaling algorithm changed drastically. The Kubernetes auto-scaling algorithm suddenly increased the number of scaled microservices and suddenly shrunk microservices. Kubernetes auto-scaling exhibited drastic changes in its scaling strategy. In an edge network with limited resources, such changes consume additional computing resources and affect the stability of the overall computing network. Compared with Kubernetes auto-scaling, the designed FMCRS scaling method was able to immediately meet the needs of microservice resource expansion and reduction, and thus, no drastic change occurred in the microservice resource management strategy. Therefore, FMCRS scaling is more suitable for the microservice resource management environment in edge computing networks.

## 6. Conclusions

In this study, we designed and implemented a prototype of a microservice resource management platform for edge computing networks. The core of the platform includes a fuzzy-based microservice computing resource scaling (FMCRS) management algorithm for microservice resource expansion based on fuzzy theory. This study also introduces the PSO algorithm for optimizing the fuzzy membership function of the proposed fuzzy microservice resource management algorithm. In addition, we designed and implemented two methods for horizontal scaling and vertical scaling in accordance with the available resources of edge computing network nodes. In this study, we conducted extensive experiments to verify the effectiveness of the proposed platform and the performance of the FMCRS algorithm. The results of the experiment proved that the proposed microservice resource management platform was able to reduce the response time of microservice resource adjustments and dynamically scale microservices horizontally and vertically. Moreover, the performance of the proposed FMCRS was similar to that of a currently widely used cloud computing microservice resource management mechanism, namely, Kubernetes auto-scaling. However, no dramatic change in the microservice resource management strategy occurred in FMCRS. FMCRS was able to avoid the sudden surge in overall network resource allocation, and thus, it is more suitable for the edge computing microservice management environment. Therefore, the system prototype designed in this study will provide users of edge computing networks with a new option for meeting the increasingly complex network application requirements.

In a future study, the proposed microservice resource management platform will be implemented as a service/software as a service (SaaS) on the various public cloud service platforms, such as AWS, GCP, and Azure. More IoT edge computing application use cases, such as smart farming and smart factory, will also be tested on our platform to see what needs to be improved. We will also study how to combine machine learning and deep learning models in our FMCRS algorithm to improve the efficiency of microservice resource management, such as reducing latency and improving the service quality.

## Figures and Tables

**Table 1:** Fuzzy rules of the microservice resource scaling management fuzzy inference system.

Rule Number	CPU Loading	Memory Loading	Scaling Extent of Computing Resource
1	High	High	High
2	Medium	High	Medium
3	High	Medium	Medium
4	Medium	Medium	Medium
5	Low	Medium	Medium
6	Medium	Low	Medium
7	Low	Low	Low

**Table 2:** Hardware specifications of the implemented fuzzy-based microservice resource management platform for edge computing.

Hardware	Specification
Server Manufacturer/Model	Dell/PowerEdge R1415
CPU	AMD Opteron™ Processor 4280
Memory	DDR-3 1333 32 GB
Disk Storage	WD20EARX 2.0 TB
Network Inference Card	Broadcom Corporation NetXtreme II BCM5716 Gigabit Ethernet
Server Operating System	Ubuntu 16.04.4
Edge Computing Nodes Computer	Raspberry Pi 3
Edge Node Sensing Device	On-Board Diagnostics (OBD) Connector

**Table 3:** Software specifications of the implemented fuzzy-based microservice resource management platform for edge computing.

Software	Specification
Resource Monitoring Database	Prometheus 1.8.2
TCP/HTTP Load Balancer	HA Proxy 1.4.27
Network Performance Measurement and Tuning	Iperf 3.1.3
Application Server	Nginx 1.13.6
Database Server	PostgreSQL 9.1

**Table 4:** Parameter settings in experiment 6.

Attributes	Values
Initial Microservice Instance	1
Maximum Microservice Experiment Time	20
Experiment Time	960 s
HTTP Request Rate Update Frequency	180 s
Maximum HTTP Request Rate	1000 bits/s
CPU Loading High Alarm Threshold	80%
Memory Loading High Alarm Threshold	80%

## Data Availability

Not applicable.
